# The effect of maternal prenatal cannabis exposure on offspring preterm birth: a cumulative meta-analysis

**DOI:** 10.1192/j.eurpsy.2023.215

**Published:** 2023-07-19

**Authors:** B. D. Adema, B. Dachew, G. Pereira, R. Alati

**Affiliations:** Curtin School of Population Health, Curtin University, Perth, Australia

## Abstract

**Introduction:**

Mixed results have been reported on the association between prenatal cannabis exposure and preterm birth.

**Objectives:**

This systematic review and meta-analysis aimed to examine the magnitude and consistency of associations reported between prenatal cannabis exposure and preterm birth.

**Methods:**

This review was guided by the Preferred Reporting Items for Systematic Review and Meta-Analysis (PRISMA) guidelines. We performed a comprehensive search of the literature on the following electronic databases: PubMed, EMBASE, SCOPUS, Psych-INFO, and Web of Science. The revised version of the Newcastle-Ottawa Scale (NOS) was used to appraise the methodological quality of the studies included in this review. Inverse variance weighted random effects cumulative meta-analysis was undertaken to pool adjusted odds ratios (AOR) after sequential inclusion of each newly published study over time. The odds ratio and 95% confidence interval (CI) limits required for a new study to move the cumulative odds ratio to the null were also computed.

**Results:**

A total of 27 observational studies published between 1986 and 2022 were included in the final cumulative meta-analysis. The sample size of the studies ranged from 304 to 4.83 million births. Prenatal cannabis exposure was associated with an increased risk of preterm birth [pooled Adjusted Odds Ratio (AOR) = 1.35, 95% CI: 1.24-1.48]. The stability threshold was 0.74 (95%CI limit 0.81) by the end of 2022.

**Conclusions:**

Offspring exposed to maternal prenatal cannabis use was associated with higher risk of preterm birth and it is strongly unlikely that any new epidemiological studies will change this conclusion. It is also plausible that avoiding cannabis intake during the prenatal period can reduce the risk of preterm birth.

**Disclosure of Interest:**

None Declared

